# Supplementary material for: Target-responsive vasoactive probes for ultrasensitive molecular imaging
Source: Nat Commun. 2020 May 13;11:2399. doi: 10.1038/s41467-020-16118-7 (PMC7220906; doi:10.1038/s41467-020-16118-7)
Supplement: Supplementary file 1 — Supplementary Information [file 41467_2020_16118_MOESM1_ESM.pdf]

Target-responsive vasoactive probes for ultrasensitive molecular imaging

Robert Ohlendorf*, Agata Wiśniowska*, Mitul Desai, Ali Barandov,
Adrian L. Slusarczyk, Nan Li & Alan Jasanoff

Supplementary Information

Table of Contents **Page**

Supplementary Methods 2

Supplementary Table 1 | PAC1 activation by PACAP variants 3

Supplementary Figure 1 | MRI signal changes arising from intracranial PACAP delivery 4

Supplementary Figure 2 | MRI signal changes arising from tandem PACAP injections 5

Supplementary Figure 3 | Comparison of vasoprobe and sensory-induced MRI signal changes 6

Supplementary Figure 4 | Use of desthiobiotin for BT-AVATar construction 7

Supplementary Figure 5 | BT-AVATar variant containing one tethered dBT 8

Supplementary Figure 6 | Biotin-labeled and control cells for implantation 9

Supplementary Figure 7 | Quantification of streptavidin binding by biotinylated cells 10

Supplementary Figure 8 | Synthesis of PACAP-10,20-DA 11

Supplementary Figure 9 | NMR spectra of DA-Mal 12

Supplementary Figure 10 | Model of DA-AVATar design parameter effects 13

Supplementary Figure 11 | DA-AVATar variant containing three tethered dopamine moieties 14

SUPPLEMENTARY METHODS

General synthesis-related information

Mal-NHS (**1**) was purchased from Matrix Scientific (Columbia, SC) and used without further purification. All other chemicals were purchased from Sigma-Aldrich. Mass spectra were recorded on Agilent (Santa Clara, CA) 6545 Q-TOF and Bruker (Billerica, MA) Omniflex MALDI-TOF instruments. Nuclear magnetic resonance (NMR) spectra were recorded on a Bruker AVANCE III-400 NMR (400 MHz).

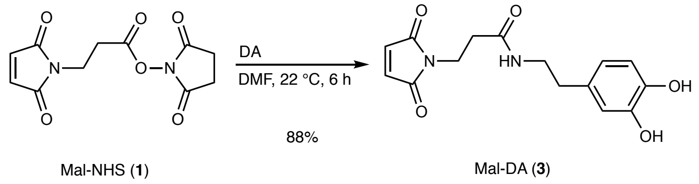

Synthesis of Mal-DA (3)

Mal-NHS (270 mg, 0.1 mmol) and dopamine hydrochloride (190 mg, 0.1 mmol) were dissolved in anhydrous dimethylformamide (DMF, 2 mL) and after addition of *N*,*N*-diisopropylethylamine (DIEA, 130 mg, 0.1 mmol) the reaction solution was stirred under argon atmosphere at room temperature for 6 h. The resulting solution was treated with deionized water (20 mL) and extracted with EtOAc (3 x 50 mL). The combined organic phases were dried over $MgSO_4$ and after removal of all volatiles the resulting residue was purified by high performance liquid chromatography (HPLC, silica-C18, eluent gradient H_2O:MeCN from 95:5 to 10:90; t_R = 8.3 min). Yield: 260 mg (88%). MS (HRESI): *m/z* = 303.0991 (theoretical) $[M-H]^-$, 303.0928 (experimental) $[M-H]^-$. ^{1}H NMR (400 MHz, DMSO-d_6) δ 7.97 (t, *J* = 5.8 Hz, 0H), 7.00 (s, 1H), 6.61 (d, *J* = 7.9 Hz, 0H), 6.55 (d, *J* = 1.6 Hz, 0H), 6.47 – 6.30 (m, 0H), 3.59 (t, *J* = 7.3 Hz, 1H), 3.12 (q, *J* = 6.9 Hz, 1H), 2.46 (t, *J* = 7.7 Hz, 1H), 2.30 (t, *J* = 7.3 Hz, 1H). ^{13}C NMR (101 MHz, DMSO) δ 170.75, 169.15, 145.03, 143.48, 134.54, 130.15, 119.13, 115.88, 115.44, 40.53, 40.15, 39.99, 39.94, 39.73, 39.52, 39.31, 39.10, 38.89, 34.55, 34.08, 34.03, 0.11.

Supplementary Table 1 | PAC1 activation by additional PACAP variants

**Construct**	**–Log{EC_{50}} –SA[a]**	**–Log{EC_{50}} +SA[a]**
PACAP	10.97 ± 0.01	10.81 ± 0.01
PACAP-10-BT	10.20 ± 0.01	8.91 ± 0.02
PACAP-10-PEG-BT[b]	9.45 ± 0.05	8.70 ± 0.01
PACAP-20-PEG-BT[b]	10.7 ± 0.1	9.30 ± 0.02
PACAP-10-BT (repurified)[c]	10.69 ± 0.01	9.20 ± 0.04

[a] All values in the absence (–SA) or presence (+SA) of streptavidin were measured in duplicate using the PAC1 receptor activation bioassay and presented as mean ± SD.

[b] These variants contain a BT-modified glutamine residue at positions 10 or 20, each with a linker of four polyethylene glycol (PEG) monomers separating the BT moiety from the glutamine sidechain.

[c] PACAP-10-BT was repurified using HPLC to remove potential contaminants.

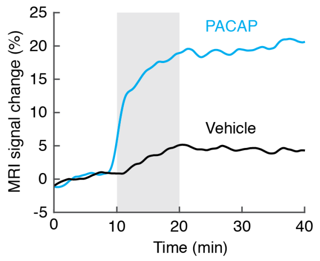

Supplementary Figure 1 | MRI signal changes arising from intracranial PACAP delivery. MRI time courses during delivery of 100 nM PACAP (blue) or aCSF vehicle (black) (n = 1).

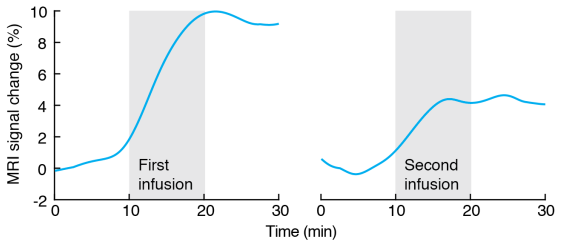

Supplementary Figure 2 | MRI signal changes arising from tandem PACAP injections. Time courses of MRI signal change near the injection region following injections of 1 µM PACAP (0.1 µL/min) over 10 minute intervals within tandem imaging experiments, analogous to main text **Figure 1c-e**. Data presented from a single animal shows responses to PACAP in both experimental periods.

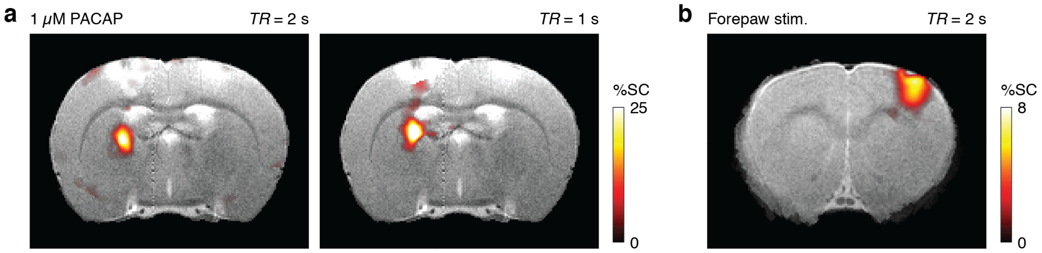

Supplementary Figure 3 | Comparison of vasoprobe and sensory-induced MRI signal changes. (a) Map of MRI signal change following intracranial delivery of 1 μM of activated BT-AVATar (PACAP-10,20,32-dBT) imaged with *TR* = 2 s (left) and *TR* = 1 s (right), with no temporal averaging, overlaid on an anatomical scan (n = 1). **(b)** Map of mean MRI signal change following forepaw stimulation in an individual animal imaged with *TR* = 2 s and other parameters identical to those in panel **a** (n = 1).

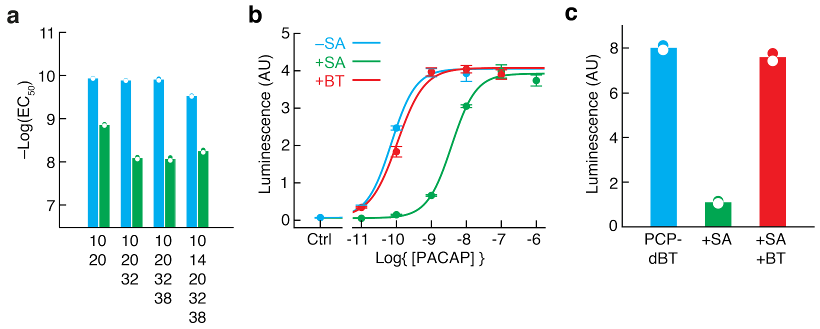

Supplementary Figure 4 | Use of desthiobiotin for BT-AVATar construction. (a) EC_{50} values for activation of PAC1 by PACAP derivatives modified with desthiobiotin (dBT) at the residue positions shown, in the absence (blue) and presence (green) of 200 nM SA. **(b)** Titration of PAC1 receptor activation by PACAP-10,20,32-dBT in the absence (blue) and presence (green) of 200 nM SA, and in the presence of SA plus 8 μM biotin (BT, red). **(c)** Bioassay output in response to PACAP-10,20,32-dBT (blue) with added SA (green) and additionally added BT (red). Error bars denote SD of duplicate (panels a, c) and triplicate (panels b) measurements.

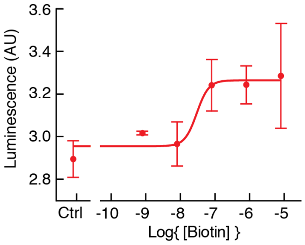

Supplementary Figure 5 | BT-AVATar variant containing one tethered dBT. Titration of BT-AVATar containing PACAP modified with tethered dBT only at position Y10, showing activation with EC_{50} of 23 ± 5 nM. Error bars denote SD of duplicates.

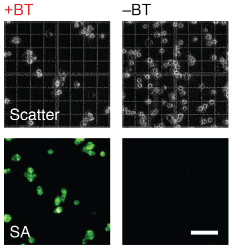

Supplementary Figure 6 | Biotin-labeled and control cells for implantation. Microscopy of biotin-labeled (+BT) and control-treated (–BT) HEK cells by scattered light (top) for visualization of all cells and by SA-Dylight488 (bottom) for visualization of biotin labeling. These cells are equivalent to cells implanted and imaged using BT-AVATar in the experiments of main text **Figure 4**. Scale bar = 100 μm. Experiment was performed twice.

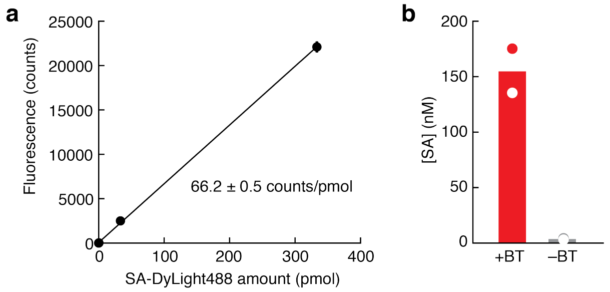

Supplementary Figure 7 | Quantification of streptavidin binding by biotinylated cells. (a) Calibration curve showing raw fluorescence output as a function of SA-DyLight488 quantity, yielding the calibration constant indicated. **(b)** Quantification of biotin concentration in cell pellets computed using the calibrated fluorescence assay based on SA-DyLight488 binding to biotin-labeled (+BT, red) or control-treated (-BT, gray) cells. Error bars denote SD of duplicates.

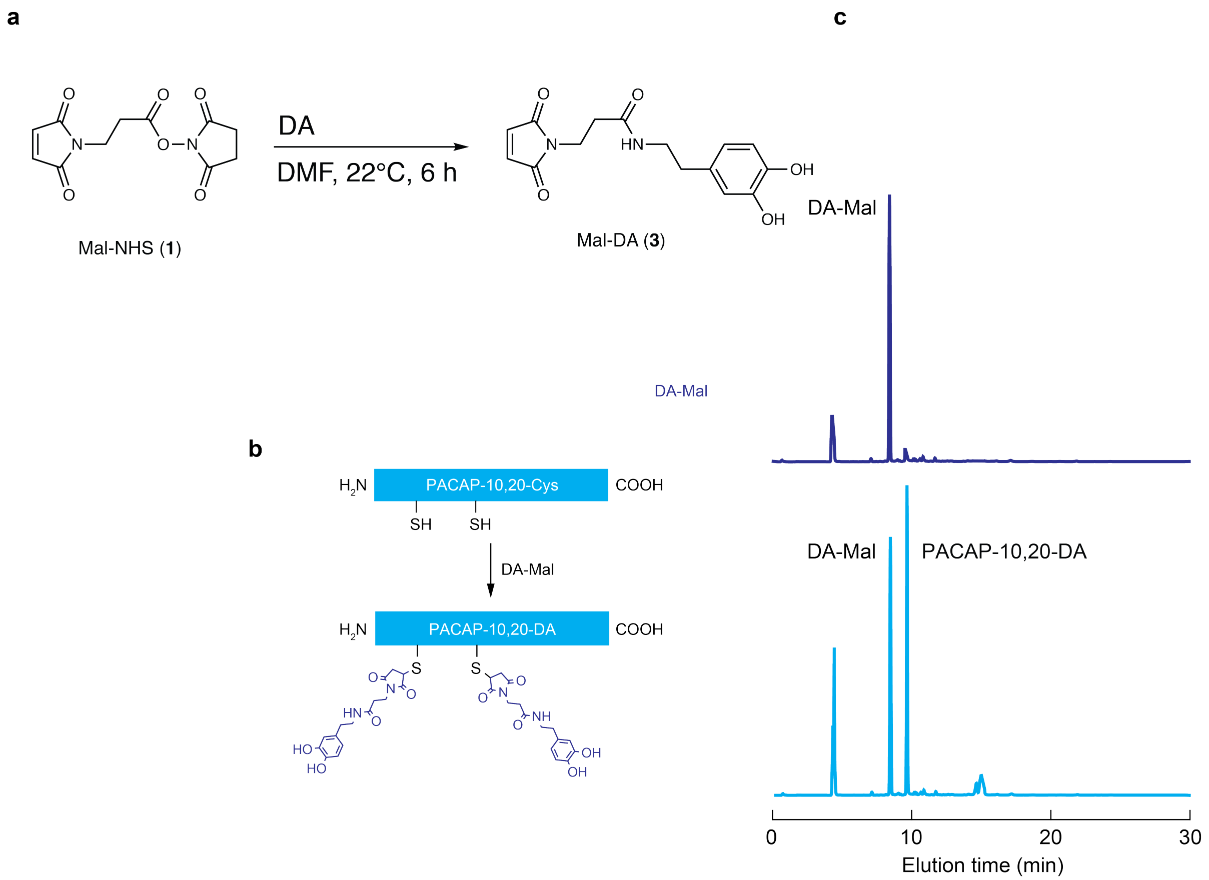

Supplementary Figure 8 | Synthesis of PACAP-10,20-DA. (a) Formation of the dopamine-maleimide conjugate DA-Mal by reaction of N-succinimidyl 3-maleimidopropionate (left) with dopamine in DMF. **(b)** The double cysteine mutant of PACAP, PACAP-10,20-Cys was synthesized by solid phase methods and reacted with excess DA-Mal to produce PACAP-10,20-DA as shown. **(c)** HPLC traces showing elution profiles of DA-Mal and PACAP-10,20-DA, following the reaction in **b**.

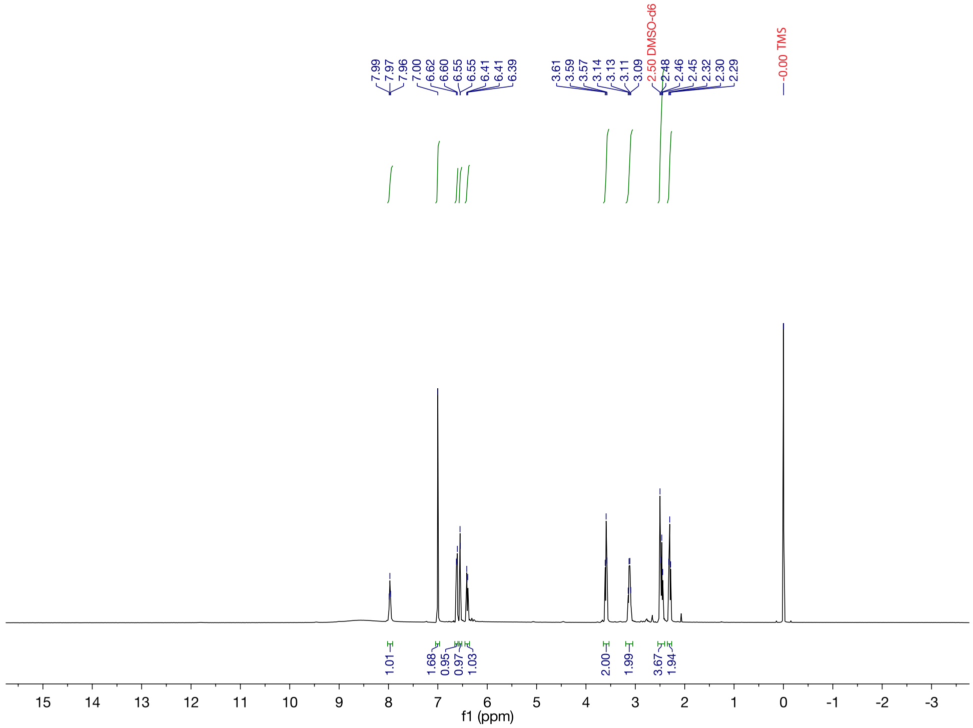

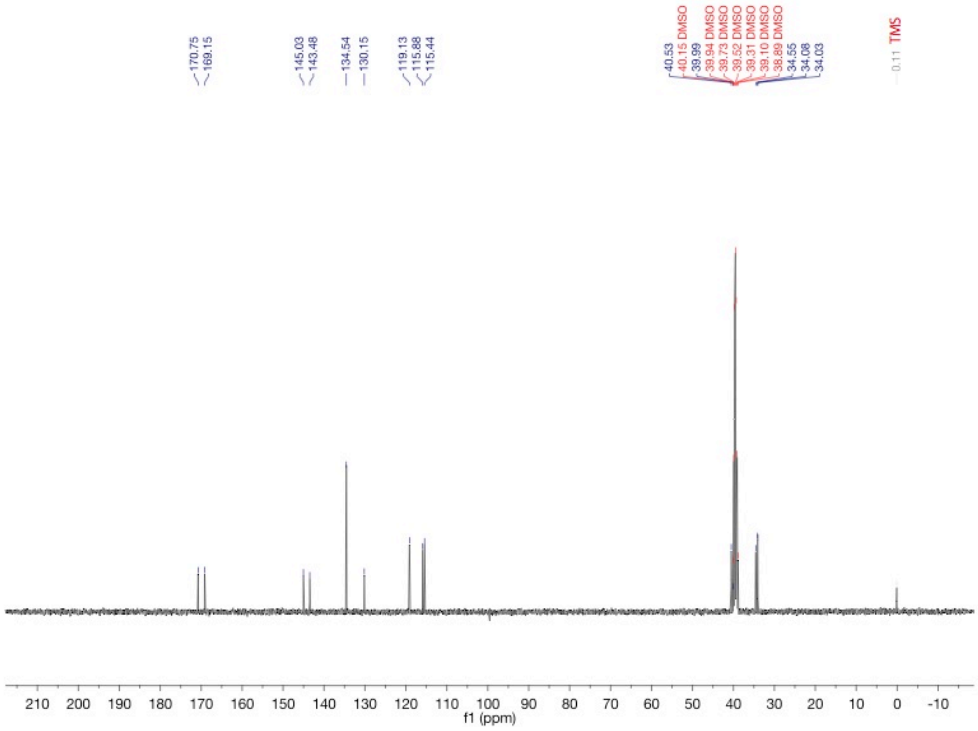

Supplementary Figure 9 | NMR spectra of DA-Mal. Top: ^{1}H; bottom: ^{13}C.

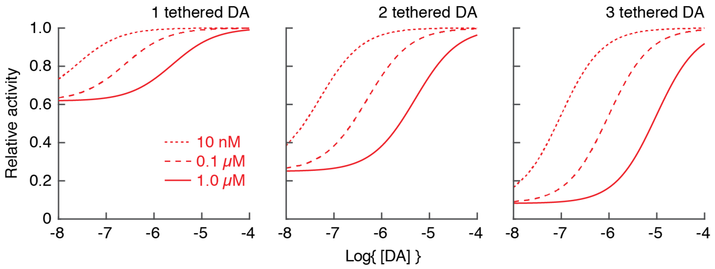

Supplementary Figure 10 | Model of DA-AVATar design parameter effects. Results of a simple model that shows how multiple design parameters can affect performance of DA-AVATars. An equilibrium model of probe activation assumes a PACAP moiety concentration of 10 nM modified by one (left), two (middle), or three (right) tethered DA groups. An IgG-based blocking component at a concentration equal to the number of tethered DA moieites is assumed to bind each tethered DA independently, with a dissociation constant (K_d) of 10 nM. This binding is in competition with nondepleting binding of DA, according to varying K_d values as indicated by the key at left. Each modeled DA-AVATar is assumed to be active when no IgG domains are bound, but inactive if one or more IgG is bound. Both the assumption of independent IgG binding and all-or-nothing functionality of the probe are simplifications, but the modeled titration curves nevertheless indicate that both the K_d values and number of tethered DA moieties together influence the EC_{50} and dynamic range of probe performance.

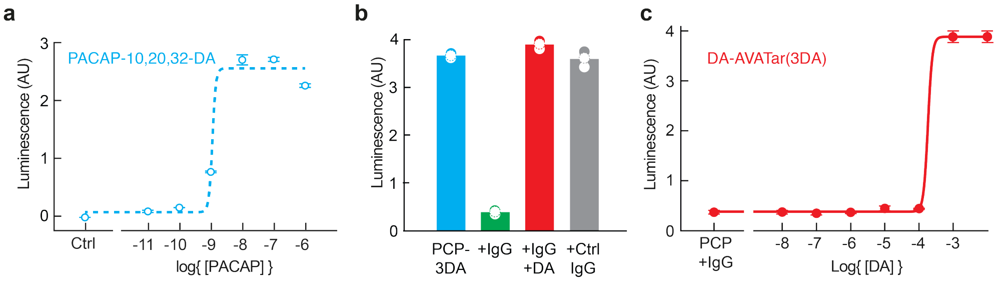

Supplementary Figure 11 | DA-AVATar variant containing three tethered dopamine moieties. (a) Titration of PACAP-10,20,32-DA in the PAC1 cell-based bioassay. **(b)** PAC1 receptor activation by 1 nM PACAP-10,20,32-DA in the absence (blue) or presence (green) of 600 nM DA-binding IgG. Activity is restored by addition of excess DA (red). Control IgG (gray) does not block PACAP activity. **(c)** Titration of the DA response of 1 nM DA-AVATar formed from PACAP-10,20,32-DA in the PAC1 activation assay. DA activates this DA-AVATar with an EC_{50} of 184 ± 7 μM. Error bars denote SD of duplicates (panel a) or triplicates (panels b, c).
