# Supplementary material for: Target-responsive vasoactive probes for ultrasensitive molecular imaging
Source: Nat Commun. 2020 May 13;11:2399. doi: 10.1038/s41467-020-16118-7 (PMC7220906; doi:10.1038/s41467-020-16118-7)
Supplement: Supplementary file 2 — Reporting Summary [file 41467_2020_16118_MOESM2_ESM.pdf]

nature research

Corresponding author(s): Alan Jasanoff

Last updated by author(s): Mar 19, 2020

Reporting Summary

Nature Research wishes to improve the reproducibility of the work that we publish. This form provides structure for consistency and transparency in reporting. For further information on Nature Research policies, see Authors & Referees and the Editorial Policy Checklist .

Statistics

For all statistical analyses, confirm that the following items are present in the figure legend, table legend, main text, or Methods section.

n/a	Confirmed	
☐	☒	The exact sample size (*n*) for each experimental group/condition, given as a discrete number and unit of measurement
☐	☒	A statement on whether measurements were taken from distinct samples or whether the same sample was measured repeatedly
☐	☒	The statistical test(s) used AND whether they are one- or two-sided<br>*Only common tests should be described solely by name; describe more complex techniques in the Methods section.*
☒	☐	A description of all covariates tested
☒	☐	A description of any assumptions or corrections, such as tests of normality and adjustment for multiple comparisons
☐	☒	A full description of the statistical parameters including central tendency (e.g. means) or other basic estimates (e.g. regression coefficient) AND variation (e.g. standard deviation) or associated estimates of uncertainty (e.g. confidence intervals)
☐	☒	For null hypothesis testing, the test statistic (e.g. *F*, *t*, *r*) with confidence intervals, effect sizes, degrees of freedom and *P* value noted<br>*Give P values as exact values whenever suitable.*
☒	☐	For Bayesian analysis, information on the choice of priors and Markov chain Monte Carlo settings
☒	☐	For hierarchical and complex designs, identification of the appropriate level for tests and full reporting of outcomes
☒	☐	Estimates of effect sizes (e.g. Cohen's *d*, Pearson's *r*), indicating how they were calculated

Our web collection on statistics for biologists contains articles on many of the points above.

Software and code

Policy information about availability of computer code

Data collection	Paravision 6.0.1
Data analysis	Matlab R2018b, Prism 8, AFNI 18.2.15

For manuscripts utilizing custom algorithms or software that are central to the research but not yet described in published literature, software must be made available to editors/reviewers. We strongly encourage code deposition in a community repository (e.g. GitHub). See the Nature Research guidelines for submitting code & software for further information.

Data

Policy information about availability of data

All manuscripts must include a data availability statement. This statement should provide the following information, where applicable:

- Accession codes, unique identifiers, or web links for publicly available datasets
- A list of figures that have associated raw data
- A description of any restrictions on data availability

Raw datasets generated during and/or analyzed during the current study are available from the corresponding author on reasonable request.

Field-specific reporting

Please select the one below that is the best fit for your research. If you are not sure, read the appropriate sections before making your selection.

☒ Life sciences ☐ Behavioural & social sciences ☐ Ecological, evolutionary & environmental sciences

For a reference copy of the document with all sections, see nature.com/documents/nr-reporting-summary-flat.pdf

Life sciences study design

All studies must disclose on these points even when the disclosure is negative.

Sample size	Replicates were performed to ensure reproducibility of measurements, not to meet explicit statistical criteria. Sample sizes for in vivo experiments were sufficient to report the observed effect sizes with appropriate statistical confidence.
Data exclusions	No data was excluded.
Replication	All experiments were replicated to ensure reproducibility and enable measurement of mean effect sizes.
Randomization	Not performed.
Blinding	Not performed.

Reporting for specific materials, systems and methods

We require information from authors about some types of materials, experimental systems and methods used in many studies. Here, indicate whether each material, system or method listed is relevant to your study. If you are not sure if a list item applies to your research, read the appropriate section before selecting a response.

Materials & experimental systems

n/a	Involved in the study	
☐	☒	Antibodies
☐	☒	Eukaryotic cell lines
☒	☐	Palaeontology
☐	☒	Animals and other organisms
☒	☐	Human research participants
☒	☐	Clinical data

Methods

n/a	Involved in the study	
☒	☐	ChIP-seq
☒	☐	Flow cytometry
☐	☒	MRI-based neuroimaging

Antibodies

Antibodies used	Anti-Dopamine antibody ab6427 (Abcam, Cambridge, MA)
Validation	Antibody is commercially available and validation was performed by manufacturer and was supported by multiple publications:<br>Fink J et al. Development of a Competition-Binding Assay to Determine Binding Affinity of Molecules to Neuromelanin via Fluorescence Spectroscopy. Biomolecules 9:175 (2019).<br>Taylor-Whiteley TR et al. Recapitulating Parkinson's disease pathology in a three-dimensional human neural cell culture model. Dis Model Mech 12:dmm038042 (2019).<br>Banks DA et al. MK-STYX Alters the Morphology of Primary Neurons, and Outgrowths in MK-STYX Overexpressing PC-12 Cells Develop a Neuronal Phenotype. Front Mol Biosci 4:76 (2017).<br>Wu SF et al. Dopamine modulates hemocyte phagocytosis via a D1-like receptor in the rice stem borer, Chilo suppressalis. Sci Rep 5:12247 (2015).<br>Forati E et al. Neurotransmitter Specific, Cellular-Resolution Functional Brain Mapping Using Receptor Coated Nanoparticles: Assessment of the Possibility. PLoS One 10:e0145852 (2015).

Eukaryotic cell lines

Policy information about cell lines

Cell line source(s)	CHO K1 (Sigma-Aldrich, St. Louis, MO), HEK293FT (Life Technologies, Grand Island, NY)
Authentication	All cell lines are commercially available and validation was performed by manufacturer.
Mycoplasma contamination	Cell lines tested negative for mycoplasma contamination in the MycoAlert assay (Lonza, Walkersville, MD, USA).
Commonly misidentified lines (See ICLAC register)	No commonly misidentified cell lines were used.

Animals and other organisms

Policy information about studies involving animals; ARRIVE guidelines recommended for reporting animal research

Laboratory animals	All experiments were performed with male Sprague-Dawley rats, age 7–9 weeks, supplied by Charles River Laboratories (Wilmington, MA). Seventeen rats were used for in vivo imaging experiments described here.
Wild animals	No wild animals were used in the study.
Field-collected samples	No field samples were collected in the study.
Ethics oversight	All animal procedures were conducted in accordance with National Institutes of Health guide-lines and with the approval of the MIT Committee on Animal Care (protocol number 0718-068-21).

Note that full information on the approval of the study protocol must also be provided in the manuscript.

Magnetic resonance imaging

Experimental design

Design type	Event-related response (event = infusion of vasoprobe/AVATar)
Design specifications	Figure 1 experiment: 10 minutes baseline, followed by 10 minutes of vasoprobe infusion, followed by 20 minutes of follow-up scanning<br>Figure 4 experiment: 10 minutes baseline, followed by continuous infusion of AVATar
Behavioral performance measures	No behavioral performance measured.

Acquisition

Imaging type(s)	anatomical, functional, and T1-weighted
Field strength	7 Tesla
Sequence & imaging parameters	High resolution T2-weighted anatomical scans of each animal were obtained using a rapid ac-quisition with relaxation enhancement (RARE) pulse sequence with echo time (TE) 44 ms, re-cycle time (TR) 2,500 ms, RARE factor 8, spatial resolution 100 µm x 100 µm x 1 mm, and matrix size 256 x 256 with seven slices. Hemodynamic contrast image series were acquired using a gradient echo planar imaging (EPI) pulse sequence with TE 25 ms, TR 2,000 ms, spatial resolution 390 µm x 390 µm x 1 mm, and matrix size 64 x 64 with seven slices. T1-weighted scans were performed using a fast lowangle<br>shot (FLASH) pulse sequence with TE 5 ms, TR 93.75 ms, spatial resolution 400 !m x 400 !m x 1 mm, and matrix size 64 x 64 with seven sagittal slices.
Area of acquisition	whole brain

Diffusion MRI ☐ Used ☒ Not used

Preprocessing

Preprocessing software	Paravision, AFNI
Normalization	Spatial registration was performed. Effects were normalized to baseline and in some cases areas of the brain not affected by sensor infusion.
Normalization template	Reference animal
Noise and artifact removal	The time series data from the EPI scans were smoothed with a Gaussian spatial kernel of 1mm full-width at half-maximum prior to statistical analysis, and each voxel time course was subsequently temporally smoothed using a sliding box window of width 75.
Volume censoring	Not relevant to the study.

Statistical modeling & inference

Model type and settings	General linear model with fixed effects.
Effect(s) tested	Event-related responses, with motion correlates regressed out

Specify type of analysis: ☒ Whole brain ☐ ROI-based ☐ Both

Statistic type for inference<br>(See Eklund et al. 2016)	F-test
Correction	None

Models & analysis

n/a	Involved in the study
☒	☐ Functional and/or effective connectivity
☒	☐ Graph analysis
☒	☐ Multivariate modeling or predictive analysis
